# Valorification of crude glycerol for pure fractions of docosahexaenoic acid and β-carotene production by using *Schizochytrium limacinum* and *Blakeslea trispora*

**DOI:** 10.1186/s12934-018-0945-4

**Published:** 2018-06-16

**Authors:** Maria Bindea, Bogdan Rusu, Alexandru Rusu, Monica Trif, Loredana Florina Leopold, Francisc Dulf, Dan Cristian Vodnar

**Affiliations:** 1 Department of Food Science, Life Science Institute, University of Agricultural Sciences and Veterinary Medicine, Cluj-Napoca, Romania; 2 CENCIRA Agrofood Research and Innovation Centre, Cluj-Napoca, Romania

**Keywords:** *Blakeslea trispora*, β-Carotene, Docosahexaenoic acid, *Schizochytrium limacinum*, Crude glycerol

## Abstract

The goal of this research is the investigation of a way to maximize the production of docosahexaenoic acid (DHA) and β-carotene by optimizing the culture conditions of their sources, microalgae *Schizochytrium limacinum* and fungus *Blakeslea trispora* respectively, in a fermentation medium. The influencing factors in the fermentation process for producing DHA and β-carotene have proven to be: the concentration of carbon source (different glycerol crude and pure concentrations) for both of them, and in particular temperature for DHA and pH for β-carotene. Testing the effect of these parameters was determined: biomass, DHA and β-carotene concentration. The highest production by *S. limacinum* was obtained at 25 °C, while using a quantity of 90 g/L of glycerol (crude or pure) as a carbon source. Temperature was the main factor that influenced the biosynthesis of DHA. The quantification of DHA was made by GC–MS chromatography, followed by a purification process, with the end result of DHA in pure phase. The maximum quantities for β-carotene production were obtained with pH 7 and 60 g/L of crude glycerol. The results highlight the possibility of using crude glycerol as a low-cost substrates for growth of microalgae *S. limacinum* and of fungus *B. trispora* in order to obtain the crucial molecules: docosahexaenoic acid and β-carotene.

## Background

Glycerol, a by-product of the biodiesel industry, it is produced in excess over the past years due to the increase in energy consumption, that has emerged due to the global industrialization [1]. Due to the fact that it is found in large quantities in recent years, it is an inexpensive being an important carbon source for biotechnological applications, since is non-toxic product for human health. Used in fermentation processes as a carbon source, glycerol can be metabolized in various chemical products useful for the pharmaceutical and food industry.

The conversion of crude glycerol into value-added products, such as docosahexaenoic acid, β-carotene, citric acid, 1,3-propanediol, has become an everyday life application [2], that helps minimize the negative effects on the environment and at same time contributes at the development of a new source of essential molecules production.

DHA is an important structural component of the cell membrane in various human tissues, being a polyunsaturated omega-3 fatty acid with the role of preventing diseases such as heart disease, schizophrenia, cancer, inflammation and Alzheimer’s [3–5]. DHA is one of the most studied bioactive compounds of marine origin due to clinical evidence that highlights the importance of this fatty acid in the development of neuronal and retinal functions especially in children [6, 7]. Currently the main source of DHA is fish oil, which has become a very popular food supplement since of its health benefits. The composition and quality of this traditional source of fatty acids is the variable being influenced by species of fish and environmental factors. There is a growing awareness that the current source of DHA is in decline, moreover environmental contamination may result in contamination of fish oil with heavy metals. Thus, efforts are being made to find an alternative and sustainable source for production of DHA. Recently, microalgae have been given special attention as an alternative source of DHA, and among the many species, *Schizochytrium limacinum* has been noted for its ability to turn waste in the value-add products, such as DHA, especially when grown in culture medium with glycerol [8]. *S. limacinum* is a marine microalga, which has the ability to synthesize polyunsaturated fatty acids, and contains a large amount of DHA in the cytoplasm, but it needs a significant amount of salt to grow. The concentration of salt in the culture medium can be adjusted to the optimal amount by supplementing the medium with various concentrations of crude glycerol.

Crude glycerol, due to its high salinity, favors the growth and development of marine microorganisms. The high salinity of the crude glycerol may be a consequence of the catalyst used in the process of obtaining the biodiesel [9].

Another important chemical compound for the food and pharmaceutical industry that can be produced by converting glycerol to fermentative processes is β-carotene.

β-Carotene, a precursor of vitamin A, is one of the 600 different carotenoids, and is used in functional food, feed products, medicine, cosmetic industries [10]. The β-carotene possesses antioxidant properties (in vivo and in vitro) that have a positive effect in preventing different diseases which are caused by the changes that have appeared in lifestyle, like cancer, heart disease, diabetes, hypertension [11]. It is also known as a protective agent against brain aging and human vision. In recent years, recent studies have shown that among the microorganisms that exhibits a greater potential for β-carotene production is *Blakeslea trispora* [12, 13]. *Blakeslea trispora* is a heterothallic fungus that present a main advantage over others microorganisms, namely that it does not need any special environmental conditions for growth [14]. The carotenoid biosynthesis and cell growth are improved since the *B. trispora* possesses sufficient supply of oxygen [15].

The β-carotene that is obtained from fungus is natural and it is a mixture of* trans* and* cis* isomers, and possess anticancer activity, due to this mixture. Between the β-carotene obtained from biological source and the one obtained by chemical synthesis there is a major difference, namely the mixture of* trans* with* cis* isomers is hard to get via chemical synthesis [16, 17].

This research aims to optimizing the process of obtaining valuable products like DHA and β-carotene by using as a substrate crude glycerol and by monitoring the contribution of the main environmental stress factors. The most important stress factors which have an influence of the production of fatty acid and carotenoids are the temperature, carbon source and pH.

## Materials and methods

### Microorganisms and culture conditions

#### Cultivation of *Schizochytrium limacinum*

*Schizochytrium limacinum* SR21 (ATCC MYA-1381) used in this study was purchased from American Type Culture Collection. The cells were activate in 10 mL of artificial seawater (ASW) containing: 20 g/L glucose (VWR Chemicals, Belgium), 10 g/L yeast extract (Alfa Aesar, Karlsruhe, Germany) and 20 g/L sea salts (Sigma-Aldrich, USA), the composition of ASW being reported by Chin et al. [18]. The medium pH was adjusted to 7.5 with NaOH solution (20%) before being autoclaved at 121 °C for 15 min. *S. limacinum* was incubated at 25 °C on an orbital shaker set to 150 rpm for 48 h. After 48 h 10% (v/v) of the new culture was used to inoculate one 250 mL Erlenmeyer flask containing 50 mL of ASW. The flask was incubated in the same conditions as above; the new inoculum obtained being used for further fermentation processes.

Fermentations were carried out in a laboratory stirred-tank (vessel volume 2.9 L) bioreactor (Elecrolab, Fermac 300) equipped with a digital control unit. In this experiment, different fermentative process with two types of glycerol were performed; one with pure glycerol 80–120 g/L, 20 g/L sea salts and 5 g/L glucose and the other one with crude glycerol 80–120 g/L, 20 g/L sea salts and 5 g/L glucose. The stirred tank was sterilized at a temperature of 121 °C for 30 min. Aliquots of the fermentation liquid were taken every day to determine DHA and biomass.

The pure glycerol used in this study was purchased from Merck, while the crude glycerol was received from factories that produce biodiesel in Spain. Between crude and pure glycerol there are some difference, pure glycerol is a liquid substance viscous with a vague sweet taste with no odor or color and is hygroscopic. Instead, crude glycerol is a semi solid product with a light brown color resulted as waste of biodiesel production. In contrast, the crude glycerol can be found impurities that derived from biodiesel production (methanol, salts, soaps, heavy metal, residual fatty acids).

#### Cultivation of *Blakeslea trispora*

The two strains used throughout this investigation were *B. trispora ATCC 14271* mating type (+) and *B. trispora ATCC 14272* mating type (−) purchased from ATCC. The cultures were maintained on yeast agar at 25 °C for 7 days. After 7 days, the slants were kept at 4 °C, and thereafter sub-cultured every 30 days.

The fermentation was conducted in 1 L bioreactor at a filling volume of 700 mL of the culture medium (control medium). Experiments were performed with control medium supplemented with pure or crude glycerol in different concentrations. The shaking speed was 250 rpm.

The bioreactor were inoculated with a spore suspension of each microorganism containing 5.0 × 10^6^ spores/mL and incubated at 26 °C for 336 h. Constant working volume was achieved throughout the experiment by periodic addition of sterilized water. Aliquots (3 × 3 mL) were sampled under sterilized conditions for further analysis.

### Separation of cell biomass

Cell of *S. limacinum* from each sample were collected at every 24 h (from bioreactor) and at the end of fermentation (1 L fermenter) were harvested by centrifugation and the pellet was washed using 0.9% Na Cl solution and the biomass was determined by weighing the mass after drying at 102 °C overnight.

For determination the quantity of biomass produced by *B. trispora* an aliquot of the culture medium was filtered under reduced pressure through a Whatman no. 1 filter paper, and cells were washed with distilled water and dried at 80 °C until constant weight (about 24 h).

### Extraction and quantification

#### Extraction and quantification of docosahexaenoic acid

The total lipids (TLs) were extracted from 5 g of wet biomass. The sample was homogenized in 20 mL of methanol for 1 min with a high power homogenizer (MICCRA D-9, Germany) and 40 mL of chloroform were added, continuing the homogenization process for another 2 min. The mixture was filtered and the process was continued using the method reported by Dulf et al. [19].

#### Extraction and quantification of β-carotene

Lipids were removed from the cells after cell rupture by freezing and thawing, using liquid nitrogen, and then by manual grinding in the presence of quartz sand until complete cell breakage occurred. Cellular lipid extraction with chloroform/methanol (2:1, v/v) mixture was performed three times (each session lasted 1 h at room temperature) using the Folch method. β-Carotene was extracted from the inactivated biomass with ethanol. The wet biomass was separated from the medium by use of a Büchner funnel.

### Purification of docosahexaenoic acid from bacterial cells

#### Saponification

Cells of *S. limacinum* were harvested by centrifugation (5000 rpm, 15 min, 4 °C), the pellet was washed using 0.85% NaCl solution and stored into freezing conditions until the purification process was made. The dry substance was determined by weighing the mass after drying at 102 °C until constant weight. The entire saponification process was performed following the procedure described by Mendes et al. [20], but few modifications were made.

After the saponification process was obtained the hydroalcoholic phase, containing the soaps. The soaps are produced by the reaction of all fatty acids from the bacterial cell with the added hydroxyl during the saponification process. The hydroalcoholic phase, was acidified with 8 mL of HCl 37% to achieve pH = 1. The hydrochloric acid broke the soaps obtaining free fatty acids (FFA). The mixture was centrifuged at 6500 rpm, 4 °C, 10 min to separate the solid and liquid phase. Then the FFA was recovered using 4 hexane extractions with 30 mL, each.

#### Methylation

For GC–MS analyses, the FFA were methylated by adding 8 mL of a mixture of methanol:H_2_SO_4_ (80:1, v/v) and maintaining 1 h at 80 °C. The sample was cooled at 20 °C, then 5 mL of double distilled water and 5 mL of hexane were added to collect the methylated FFA. The upper hexane layer was removed and another 5 mL of hexane were added to recover all the methylated FFA. The hexane containing the FFA was dried with anhydrous sodium sulphate. The solvent was evaporated in a vacuum rotary evaporator at 35 °C obtaining 0.0396 g of methylated FFA, 0.5 mL of* n*-hexane were added and the sample was analyzed by GC–MS chromatography.

#### Winterization

The winterization process was made by storing the mixture (containing the methyl esters) at − 18 °C overnight. Because of the low temperature the saturated free fatty acids crystallized. The liquid phase containing the unsaturated free fatty acids was separated from the crystals, obtaining 0.0264 g.

#### Urea complexation

The unsaturated methyl esters were mixed with 0.0924 g of urea and 0.395 mL of MeOH (for each gram of fatty acids, 3.5 g of urea and 14.98 mL of MeOH), heated at 65 °C and stirred until the solution became clear. The mixture was stored overnight at 4 °C, followed by a centrifugation process at 11,000 rpm, 4 °C 15 min to separate the urea complexing from the non-urea complexing fractions. After phase separation, a volume of 0.5 mL double distilled water at 60 °C was added to both fractions and vortexed. Then 1 mL of hexane (with 0.01% BHT by mass per volume) was added to extract the methyl esters from both phases (non-complexing and urea complexing). The hydroalcoholic phase (MeOH and water) was washed three times with 0.6 mL of hexane to recover all the fatty acids.

Afterwards, the hexane was evaporated in a vacuum rotary evaporator at 35 °C obtaining 0.0062 g from non-complexing fraction and 0.0121 g from urea complexing fraction. Both samples were analyzed by GC–MS chromatography.

### Purification of β-carotene from bacterial cells

*Blakeslea trispora*, was harvested by centrifugation and a pellet was extracted rapidly with 25 mL of a mixture of petroleum ether/ethyl acetate/MeOH (1:1:1, v/v/v) following the procedure described by Bunea et al. [21]. Three or four extractions were found to be sufficient in order to extract all the carotenoids form the bacterial cells. The extracts were combined before their transfer to ether in a separation funnel, washed with saturated saline solution and dried over sodium sulfate. The ether phase was evaporated to dryness under vacuum, using a rotary evaporator at 35 °C. The evaporated residue was then dried under a stream of nitrogen. The residue was re-dissolved in 1 mL of ethyl acetate by vortexing for 1 min. Finally, carotenoids extracted from the samples, were separated using thin layer chromatography (TLC) and solid phase extraction (SPE).

#### Saponification procedure

Saponification is an effective means of removing unwanted lipids, chlorophylls, and esters present in the sample, which may interfere with the chromatographic separation and shorten the column’s life. The saponified extract was washed with saturated saline solution and distilled water, eliminating the soap and alkaline excess. The organic layer containing carotenoids was dried over anhydrous sodium sulfate and evaporated to dryness.

#### Thin layer chromatography separation of the saponified extract

Thin-layer chromatography is a well-known procedure, efficient in monitoring analyses for identification purposes. After evaporation of the solvent, the residual total extract was dissolved in a small volume of toluene and the solution diluted with petroleum until the concentration of toluene does not exceed 10%. Silica gel 60 thin layer plates with the solvent system presented below was used for separation of carotenoids according to the number of their functional (–OH) groups. The saponified carotenoid extract was applied to the plates in form of spots, following by the immersion of the plates into the tank containing petroleum ether:acetone in ratio 7:3 (v/v).

### Purification of saponified extract on solid phase extraction column (SPE)

The crude extract (according to the extraction procedure) was concentrated and re-dissolved in a small volume of toluene and the solution diluted with petroleum until the concentration of toluene does not exceed 10%; the final volume of the raw extract being about 1 mL. This solution is then subjected to a SPE column on silica gel, pore size 60 Å. Prior to sample loading, the column needs to be conditioned with 10 mL petroleum.

At first, the column was eluted with 10 mL of a mixture of petroleum:diethyl ether in a ratio 9:1 (v/v), to afford the elution of all β-carotene (including their* cis* isomers). After the elution of the β-carotene band was completed, the eluent was changed to petroleum ether:acetone in a ratio 7:3 (v/v), over 10 mL. Thus, the second fraction was removed. To elute the third fraction (lutein and their isomers), 10 mL acetone were required. All collected fractions were verified by TLC and finally analyzed by HPLC.

#### Analysis methods

The analysis method used for quantification of DHA was GC–MS Chromatogram. For GC analysis the total lipids were methylated using acid-catalysis transesterification procedure and the fatty acid methyl esters (FAMEs) were obtained. The FAMEs were analyzed by gas chromatography–mass spectrometry (GC–MS) using a PerkinElmer Clarus 600 T GC–MS (PerkinElmer, Inc., Shelton, CT, USA) equipment. The entire process (transesterification and GC–MS analysis) was carried out following the procedure described by Dulf et al. [19].

For the quantification of β-carotene the method used was HPLC. HPLC analyses for individual carotenoids were carried out on an Agilent 1200 system with DAD detector (Agilent Tehnologies, USA) using a reversed phase EC 250/4.6 Nucleodur 300-5 C-18 ec. Column (250 × 4.6 mm), 5 µm (Macherey–Nagel, Germany). The mobile phase consisted of mixtures of acetonitrile:water (9:1, v/v) with 0.25% triethylamine (A) and ethyl acetate with 0.25% triethylamine (B). The gradient started with 90% A at 0 min to 50% A at 10 min. The percentage of A decreased from 50% at 10 min to 10% A at 20 min. The flow rate was 1 mL/min and the chromatogram was monitored at 450 nm.

## Results and discussion

### Effect of carbon sources used on the production of DHA

To supplement with energy the culture medium of *S. limacinum* and *B. trispora* microorganisms during the fermentative processes, the following carbon sources were used in different concentrations: glucose as a model medium, crude glycerol and pure glycerol.

### Effect of crude and pure glycerol on the biomass formation and DHA production by S*. limacinum*

Cultivation of *S. limacinum* was performed in the bioreactor and the concentrations of the two types of glycerol (pure and crude) used were between 10 and 120 g/L.

To understand the stages of the fermentative process with different cellular behaviors and to investigate the effect of the carbon source on biomass formation, *S. limacinum* was cultured in the bioreactor for 168 h.

**Fig. 1:**
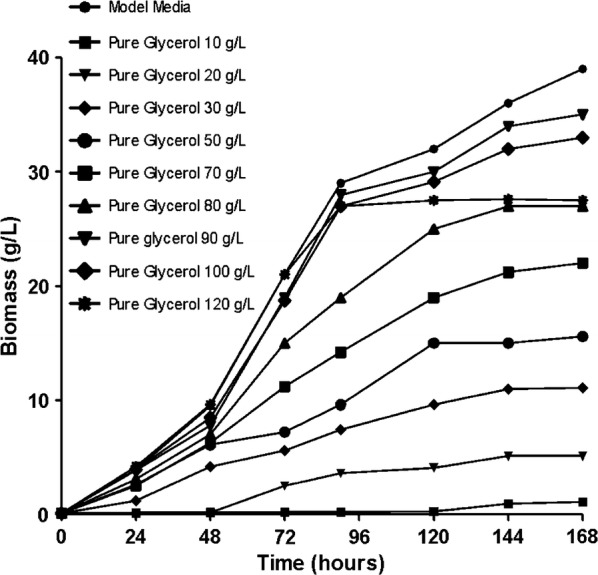
Dry biomass quantification during fermentation processes of *Schizochytrium limacinum* on pure glycerol media

**Fig. 2:**
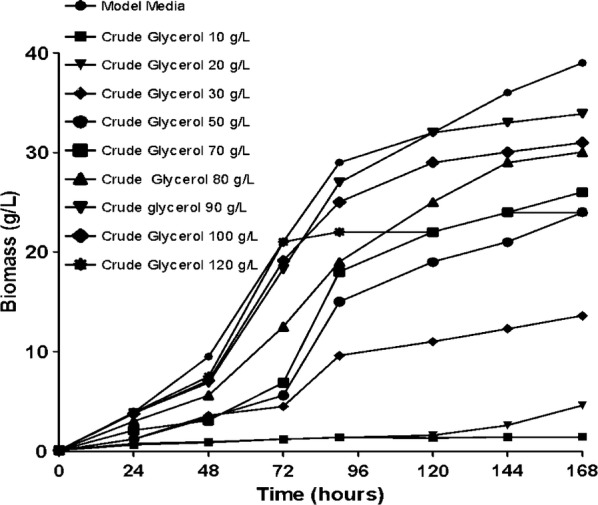
Dry biomass quantification during fermentation processes of *Schizochytrium limacinum* on crude glycerol media

Figures 1 and 2 highlight that the glycerol concentration has an effect on the amount of biomass. An improvement in the amount of biomass is observed concomitantly with an increase in glycerol concentration from 10 to 90 g/L, after which a slight decrease in biomass starts with the increase in glycerol content from 90 up to 120 g/L. These results are in according with those obtained by Patil and Gogate [22]. The biomass dry cell weight was determined by conducting a full characterization in terms of typical growth kinetics using each carbon source at regular intervals of 24 h. As shown in Figs. 1 and 2 the lowest quantity of biomass, slightly more than 0.5 g, was obtained for the crude and pure glycerol concentration of 10 g/L. The glycerol concentration of 90 g/L generated the highest amount of biomass, approximately 34.5 g/L.

The biomass productivity proved to be similar at the end of the fermentation processes carried out on the three different carbon sources used in this study.

From the experimental data can be seen that an effect on the biomass production also has the fermentation time, the biomass production increases as the fermentation process progressed. The quantity of biomass was low in the first 24 h and an increase in biomass can be observed after the initial phase. The largest amount of dry biomass was obtained after 168 h at the end of the log phase.

The concentration of glycerol in the culture medium besides it influencing the formation of biomass has an effect on the synthesis of saturated fatty acid DHA.

**Fig. 3:**
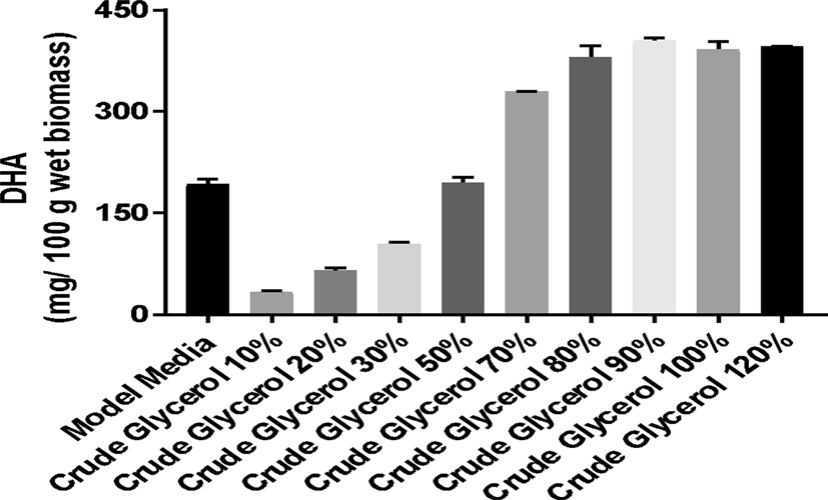
Quantification of DHA on media containing crude glycerol

**Fig. 4:**
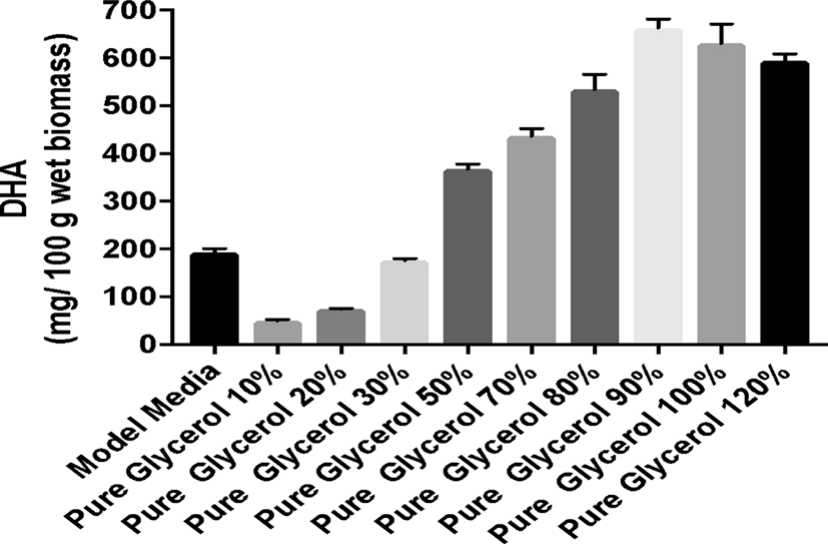
Quantification of DHA on media containing pure glycerol

Figures 3 and 4 show that DHA content gradually improved with increased glycerol concentration to 90 g/L, after which there was a slight decrease. This result is similar to that obtained in the case of biomass formation and is consistent with the literature, which reported that the optimal concentration of glycerol for production of DHA is 90 g/L [8].

On the basis of the results obtained, it can be seen that although the amount of biomass produced was similar in all three fermentation processes, the amount of DHA was higher when glycerol was used as carbon source. This increase in the amount of DHA in culture medium with crude glycerol was also observed by Patil and Gogate [22].

The final DHA concentration 598 mg/100 wet biomass was registered in trial with 100 g/L pure glycerol being lower than in trial with 90 g/L where the DHA concentration was 623 mg/100 wet biomass.

The negative effect of the too high concentration of glycerol on biomass could be attributed to the too high osmotic pressure imposed by glycerol, which has resulted in limited cell growth and product synthesis [23]. The effect of the initial amount of pure and raw glycerol on improving the production of DHA was investigated with a glycerol concentration ranging from 10 to 120 g/L (Figs. 3, 4). As can be noted in experimental data the productivity was affected by the glycerol concentration, the lowest amount was generated of a concentration 10 g/L. A better productivity can be note in the case of pure glycerol where the amount of DHA increase from approximately 50 mg/100 g (10 g/L of glycerol) at around 180 mg/100 g wet biomass (WB) when the concentration of carbon source is 30 g/L. At the concentration of 50 g/L the quantity of DHA is around of 360 mg/100 g WB when was used pure glycerol and around of 200 mg/100 g WB in case of curd glycerol. The amount of DHA obtained using pure glycerol at a concentration of 70 g/L is almost similar, approximately 400–420 mg/100 g WB, with the amount obtained in the fermentation process where the carbon source used was crude glycerol at a concentration of 90 g/L. The highest productivity was obtained when the carbon source used was pure glycerol in a concentration of 90 g/L (623 mg/100 wet biomass). When the raw and pure glycerol concentration was increased above 90 g/L, in DHA productivity, a slight decrease was observed. Thus we can say that for a high biomass production, the optimal concentration of glycerol in the culture medium is 90 g/L.

As can be seen in Figs. 1 and 2, the experimental data obtained demonstrated that glycerol can be used as a source of carbon in fermentative processes. This byproduct can successfully replace glucose, thereby reducing the culture costs of the *S. limacinum* microorganism in order to obtain biomass and, implicitly, omega 3-DHA fatty acid.

### The effect of crude and pure glycerol on the biomass formation and β-carotene production by *B. trispora*

During the fermentation process, carbon sources, in our case, crude glycerol and pure glycerol, act not only as a major constituent in the formation of cellular material but also as an important source of energy. The concentrations of the two types of glycerol used were between 10 and 180 g/L.

In order to examine the ability to convert crude and pure glycerol into β-carotene, the *B. trispora* fungus was grown in bioreactors for 336 h.

The growth of *B. trispora* in fed-batches with glucose, as a model medium, and on crude and pure glycerol in various concentrations was monitored with respect for the formation of biomass and β-carotene production.

**Fig. 5:**
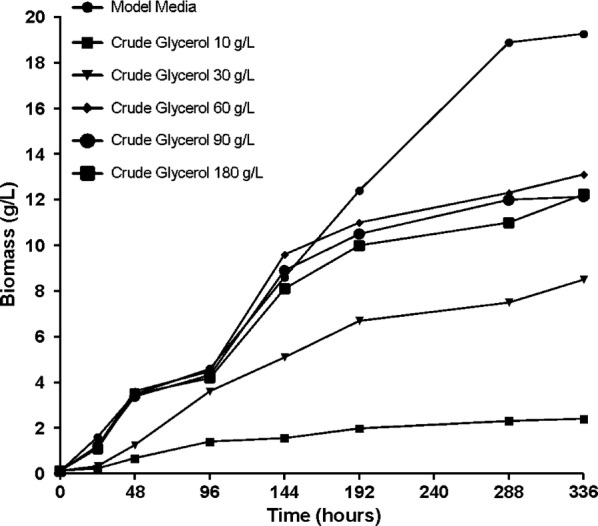
Biomass formulation during fermentation processes on media containing crude glycerol

**Fig. 6:**
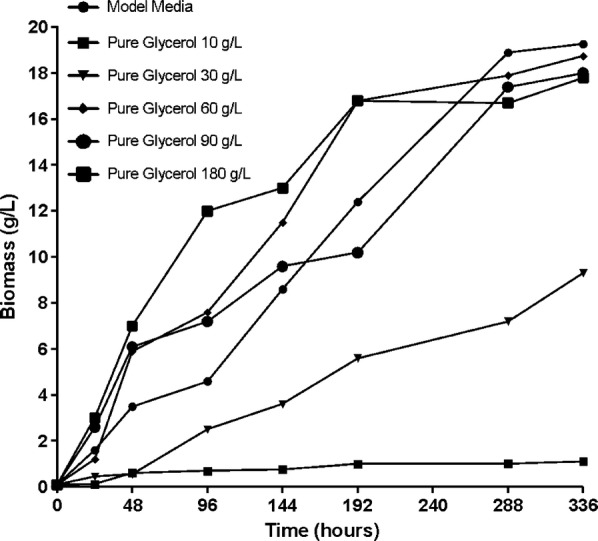
Biomass formulation during fermentation processes on media containing pure glycerol

Figures 5 and 6 represent the growth curves obtained during the fermentation process for *B. trispora*. The curves represent the amount of biomass produced depending on the growing time and the amount of glycerol to which the culture medium is supplemented.

It can be noticed that in the first 24 h there has been a low productivity of the biomass was obtained during this time for all media. For the medium supplemented with crude glycerol, the highest amount obtained during this period of fermentation was approximately 1.6 g/L for concentrations of 60, 90, 180 g/L, and in the medium supplemented with pure glycerol the amount was slightly higher compared with that obtained on crude glycerol, with a maximum of about 3 g/L for concentrations of 90 g/L, 180 g/L. Noteworthy is that although in the first 24 h in the medium supplemented with pure glycerol a higher amount of biomass is obtained for concentrations higher than 60 g/L as the cultivation time increases the best the amount of biomass will be at the concentration 60 g/L.

The experimental results show an increase in the amount of biomass in all culture media, but it was very obvious that this increase is directly proportional to the concentration of added glycerol. As can be seen in the figures there is a gradual increase of biomass with increasing the amount of glycerol to the concentration of 60 g/L, after which a slight decrease occurs.

**Fig. 7:**
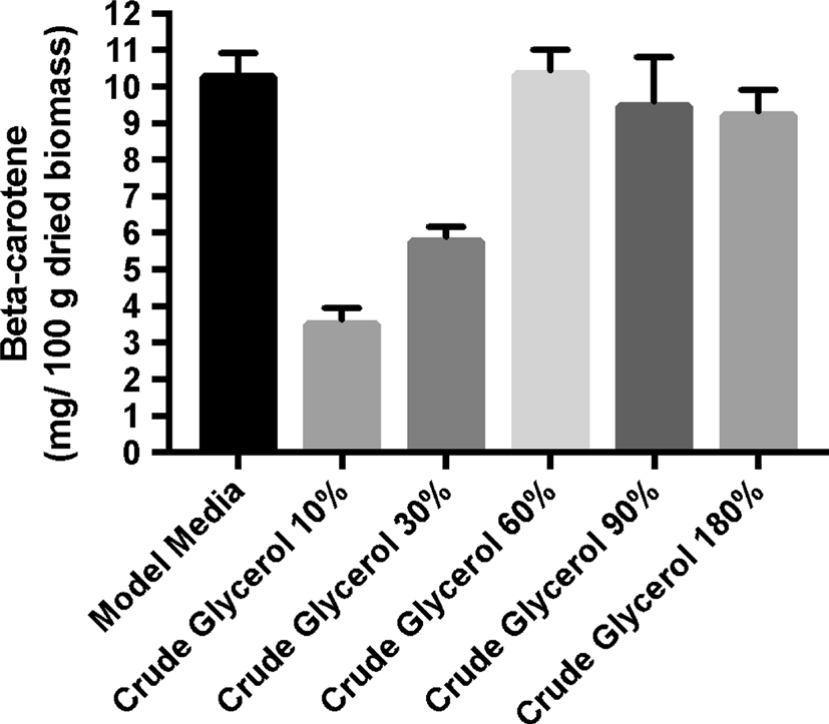
β-Carotene production during fermentation processes on media containing crude glycerol

**Fig. 8:**
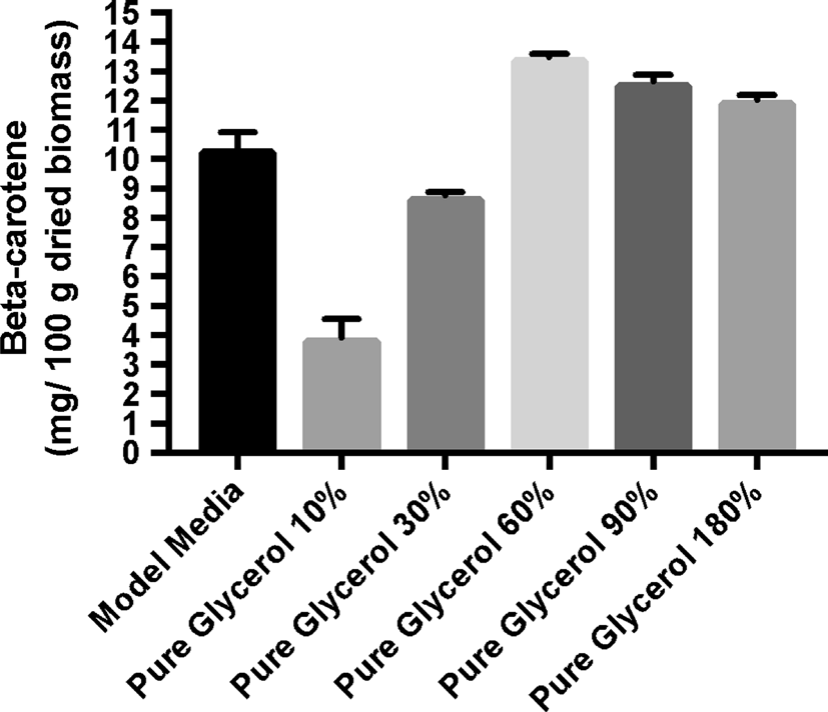
β-Carotene production during fermentation processes on media containing pure glycerol

The amount of β-carotene obtained at the end of the fermentation process during which the carbon source concentration was controlled is shown in Figs. 7 and 8.

The results presented in Figs. 7 and 8 indicate that the β-carotene production was strongly influenced by the carbon source concentration. β-Carotene production increased when the glycerol concentration was elevated from 10 to 60 g/L, after which there was a decline in β-carotene amount with the increase in glycerol concentration.

Consistent with the results obtained on determination of dry biomass, the highest amount of β-carotene was also obtained at the same concentration of glycerol (60 g/L).

### Optimizing the culture medium to improve the production of biomass and DHA by *S. limacinum*

In order to further optimize the production of biomass next to the effect of the carbon source, the effect of the temperature on cell growth and product synthesis was also investigated.

To observe the influence of temperature on the production of DHA *S. limacinum* was grown batch bioreactors at various temperatures ranging from 15 to 40 °C.

Temperature is the most important external factor that affects the growth and development of *S. limacinum*. Increasing the temperature during the fermentation process from 20 to 30 °C brought about changes in the amount of biomass accumulated but also in the biosynthesis of omega-3 DHA fatty acid.

**Fig. 9:**
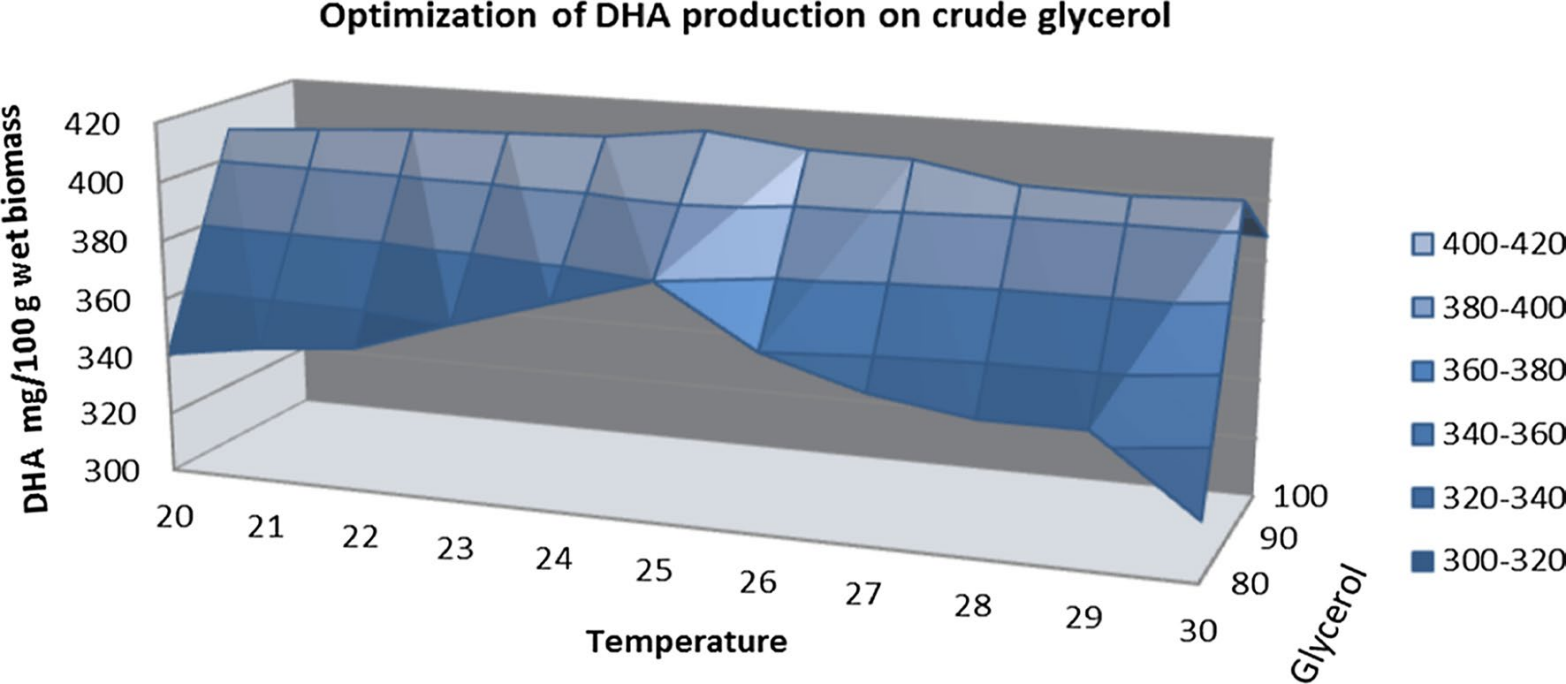
DHA optimization of media with crude glycerol

**Fig. 10:**
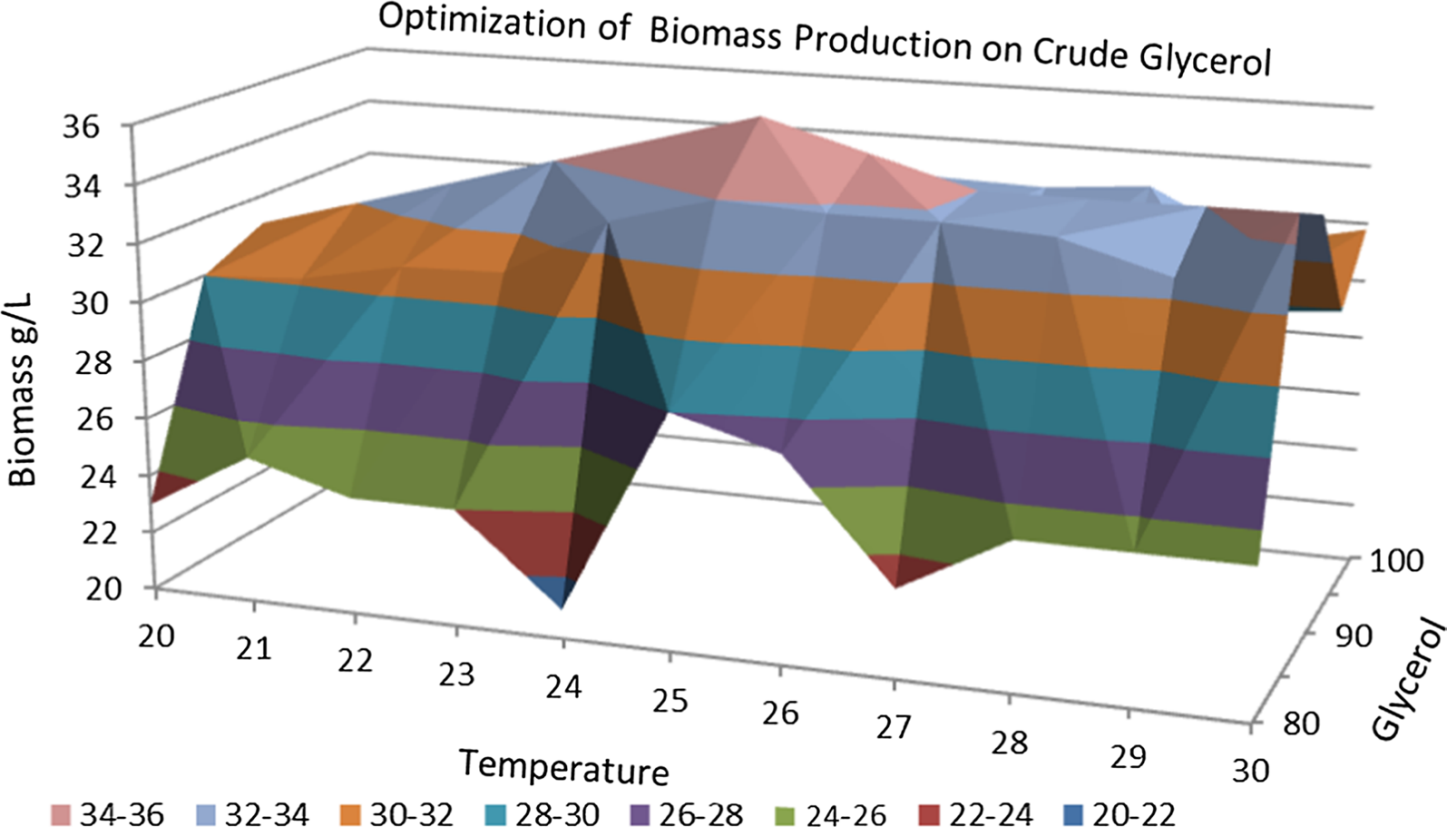
Optimization of biomass production by *S. limacinum* on crude glycerol media

**Fig. 11:**
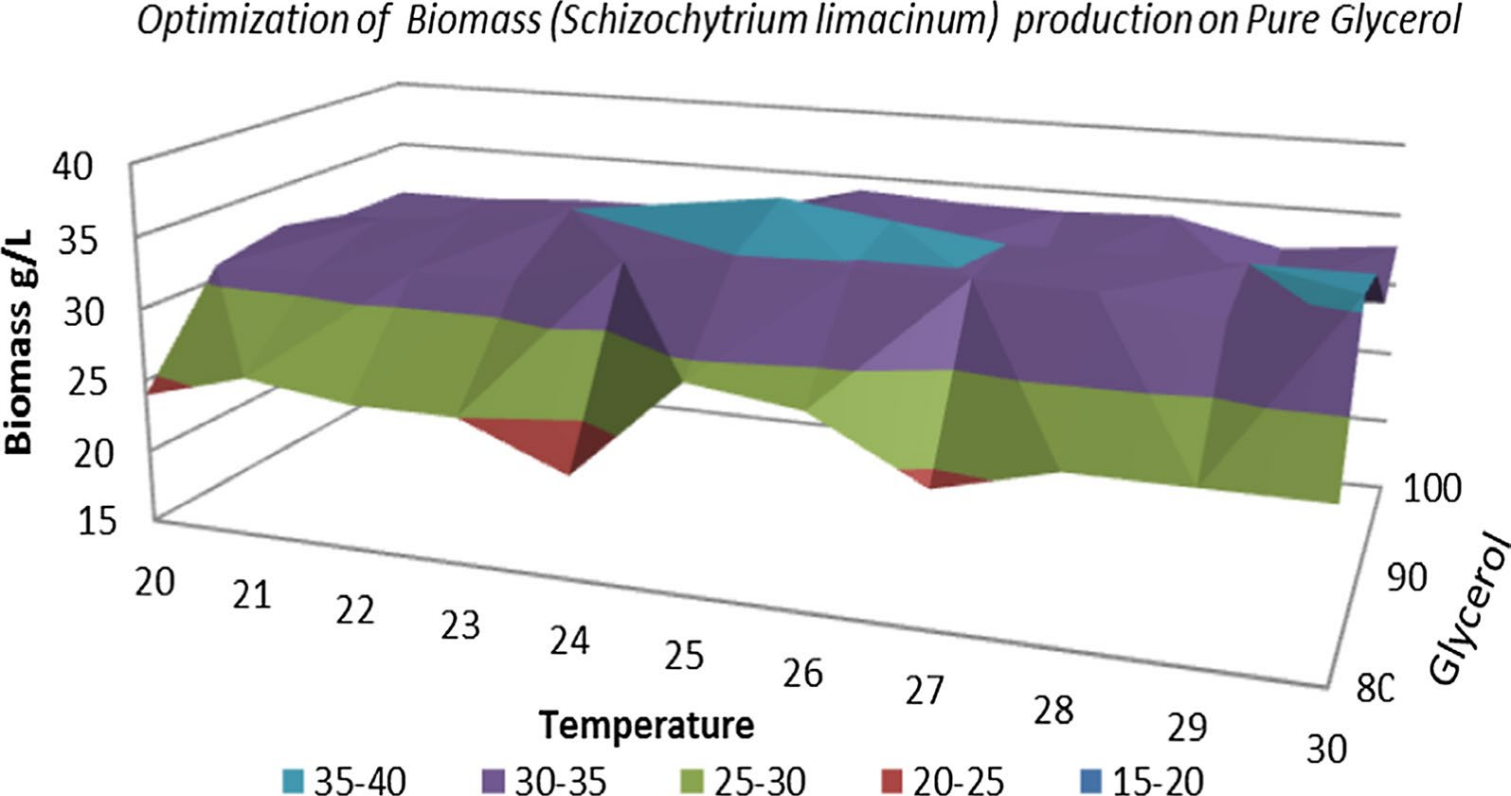
Optimization of biomass production by *S. limacinum* on pure glycerol media

In the case of biomass formation and DHA production by *S. limacinum* small changes can be observed between 20 and 24 °C and between 27 and 30 °C. The highest amount of DHA was obtained at a temperature of 25 °C, being correlated with the amount of biomass; this result is also highlighted in Figs. 9, 10 and 11. This experimental data obtained are according with those reported in literature by Chin et al. [18], which obtained a similar result when the fermentation process was carried out at 25 °C [18].

A temperature of 25 °C favored the biomass accumulation and DHA production and the explanation might be due to the increased membrane fluidity as cell response to the low temperature conditions. Also, the 25 °C led to an increased availability of intracellular molecular oxygen, which facilitates the oxygen dependent enzymes in the desaturation and elongation of DHA [5].

Small changes observed in the range 20–24 °C may be due to the fact that a lower temperature is favorable for the production of DHA, but it leads to a decrease in cell growth. The temperature optimization process must take into account both aspects, meaning the optimum temperature for increased production of DHA and the optimum temperature for cell growth, in order to maximize production of DHA [24].

Increasing the temperature to 30 °C there was a decrease in the amount of DHA. This suggests that cells adapt to a high temperature by producing more saturated fat. In order to maintain adequate lipid fluidity of the membrane at low temperatures, the proportions of unsaturated fatty acids is increased, but the highest increase can be seen with polyunsaturated acids. This is a phenotypic adaptation of the fatty acid composition in concordance with the temperature increase. Higher quantity of polyunsaturated fatty acids at low temperatures derived from lipid stabilization at these temperatures. Also increased production of polyunsaturated fatty acids at low temperatures may also be due to the sensitivity of the biosynthetic enzymes of fatty acids to temperature [24].

### Optimizing the culture medium to improve the production of biomass and β-carotene by *B. trispora*

**Fig. 12:**
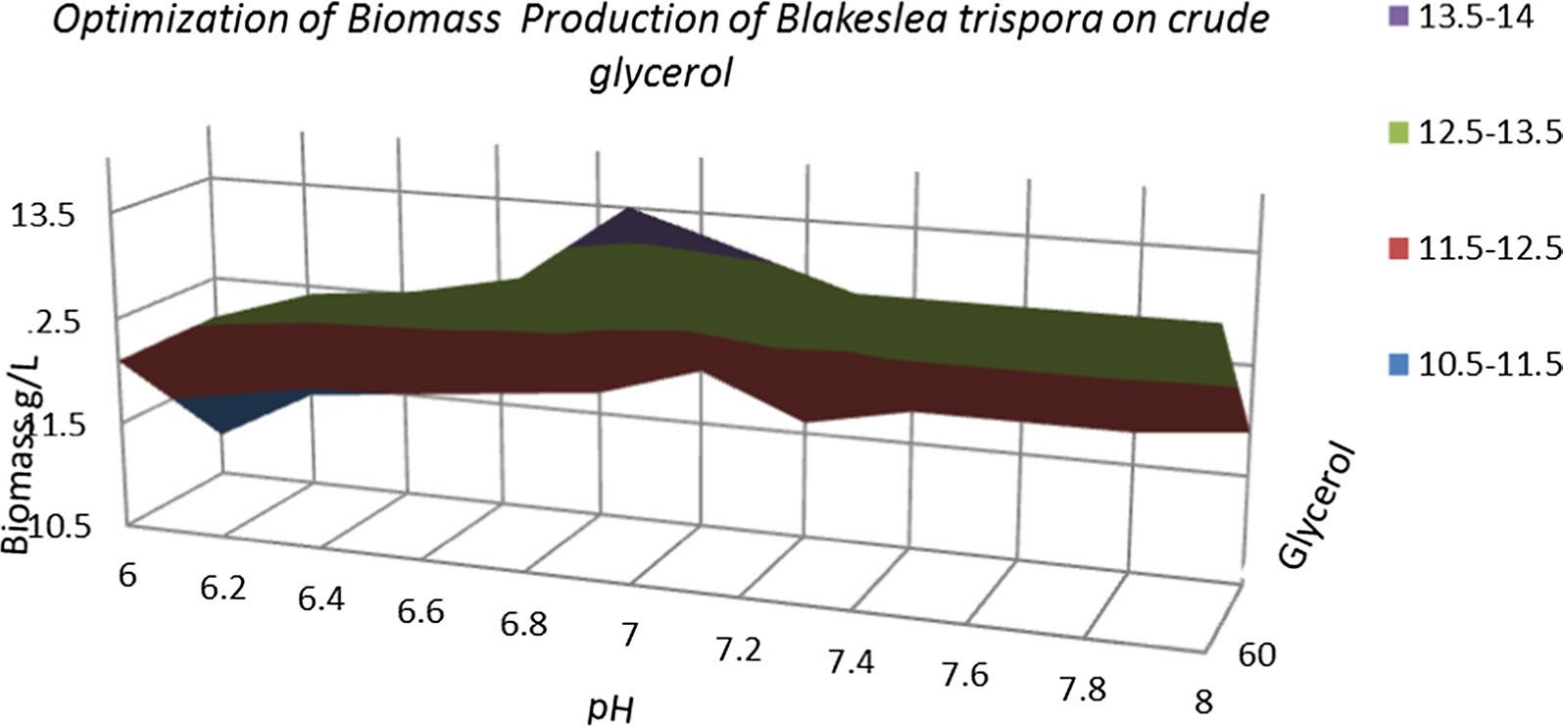
Optimization of biomass production by *B. trispora* on crude glycerol media

In an attempt to improve the production of β-carotene by cultivating *B. trispora* in the bioreactor in addition to the influence of carbon concentration, the effect of pH was also analyzed. The pH of the culture medium was controlled during the fermentation process, it was increased from 6 to 8. Figure 12, represent the amount of biomass after the optimization processes by controlling the carbon source and the pH. Regarding the biomass production of *B. trispora* in culture medium with crude and pure glycerol suggested that at the pH of 6.2 the microorganism registered the lowest biomass content (10.5–11.5 g/L) while the highest content (13.5–14 g/L) was at the pH of 6.9–7. At the 90 g/L crude glycerol fermentation, the biomass quantity was lower than in the trial which used 60 g/L crude glycerol.

Similar with biomass, the best β-carotene production was obtained at a concentration of 60 g/L of crude glycerol and at pH 7.

An important role in producing metabolites in cells has pH. The optimal pH of the culture medium in generally differs from one microorganism to another, for example the yeast *Rhodotorula acheniorum* produced a highest amount of β-carotene at a lower pH (about 5.5), in while the optimum pH for the best β-carotene production by *Serratia marcescens* RB3 is 6 [25]. Our results are in according with literature, proving that *B. trispora* produced the highest amount of biomass in an alkaline culture medium, due to the pellet formation with reduced mass transfer limitation [13].

Based on the experimental results obtained and highlighted in Fig. 12 we can conclude that *B. trispora* appeared suitable as potential strain for carotenoid production from glycerol.

### The profile of fatty acids produced by *S. limacinum* and the purification of the DHA fraction

Analyzing the profile of fatty acids produced by *S. limacinum* by the GC–MS method, we could observe the presence of the following more important fatty acids, namely: DHA (C22:6), myristic (C14:0), pentadecanoic (C15:0) and palmitic (C16:0). The experimental results reveal that DHA is the major fatty acid obtained, accounting for 36.06% of the total fatty acids. The results are consistent with those reported in the literature and indicate that *S. limacinum* has a simple fatty acid profile and the main compound is DHA [22, 26, 27].

The composition of the identified fatty acids is shown in Table 1 compared to the compositions reported in the literature. The results presented for this study in Table 1 was obtained in medium with crude glycerol concentration of 90 g/L.

**Table 1:** Fatty acid composition of *Schizochytrium limacinum* SR21

References	Fatty acid composition (%w/w of total fatty acids)			
	Myristic (C14:0)	Palmitic (C16:0)	Pentadecanoic (C15:0)	DHA (C22:6)
[28]	8.23	25.02	nr	43.42
[26]	2.7	34.2	7.6	43.1
[5]	5.26	54.70	nr	33.62
[18]	nr	55.9	nr	32.7
This study (medium-crude glycerol 90 g/L)	5.18	37.63	7.16	36.06

*nr* not reported

The differences seen in Table 1 may be due to the culture conditions in which this microalga was grown Ashford et al. [27] reported that the fatty acid profile produced by *S. limacinum* can vary depending on the components of the culture medium. Culture conditions have an effect on the ratio of triacylglycerols to phospholipids in the lipid fraction [27].

In order to purify the DHA fraction from the total lipid extract, a winterization process followed by the complex urea step was carried out. This procedure is essential for improving the DHA fraction purity, according to Ganga et al. [29]. The results obtained before winterization step regarding the DHA fraction was 36.79% and after winterization was 65%. As expected, the fraction of DHA obtained in the non-urea complex was much higher than that obtained in the winterization step.

### Purification of the β-carotene fraction

The saponified β-carotene extract before being analyzed by HLC was separated by both the TLC technique and on solid phase extraction column. A method of extracting beta carotene and lutein from the microorganisms, in which it is included the saponification step was used by Chan et al. [30].

HPLC analysis of β-carotene from *B. trispora* shows a peak at the retention time of 15.2, which reveals its presence in the sample.

Following HPLC analysis of the fractions obtained after TLC and SPE separation the β-carotene (the major compound in *Blakeslea trispora*) was purified with a yield higher than 99% (99.9%), the results are presented in Table 2. The production of β-carotene from microalga *Scenedesmus obliquus* CNN was obtained with a purity of 91.5 by the Chan et al. while from microalgae *Dunalieilla salina* was obtained with a purity of 89.4% [30, 31]. One reason for obtaining the better result may be the chosen microorganism.

**Table 2:** Percental assessment of β-carotene and lutein purification by TLC and SPE

Carotenoids	Total β-carotene of TLC fraction (%)	*Cis* isomers % of TLC fraction (%)	Total lutein after SPE separation (%)	*Cis* isomers % of SPE fraction (%)
β-Carotene	99.9	38.83	99.9	31.53
Lutein	93.7	45.9	90	31

The separation of β-carotene from *B. trispora* fungus consists of several operations involving extraction, saponification and preparative for HPLC and UV–Vis spectrophotometer respectively. Our results highlights that β-carotene can be purified with substantial success compared to literature where the β-carotene was obtained with a lower purity [30, 31].

In order to highlight the purity of β-carotene, the fractions of SPE and TLC were analyzed by UV–Vis.

The characteristic UV–Vis spectrum of β-carotene (green) and lycopene (red) reveals the high purity of this fraction, represented in Fig. 14.

**Fig. 13:**
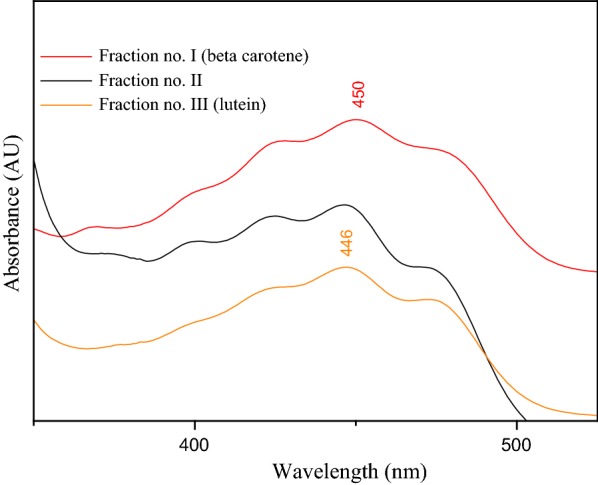
UV–Vis spectra of the SPE separated fractions

**Fig. 14:**
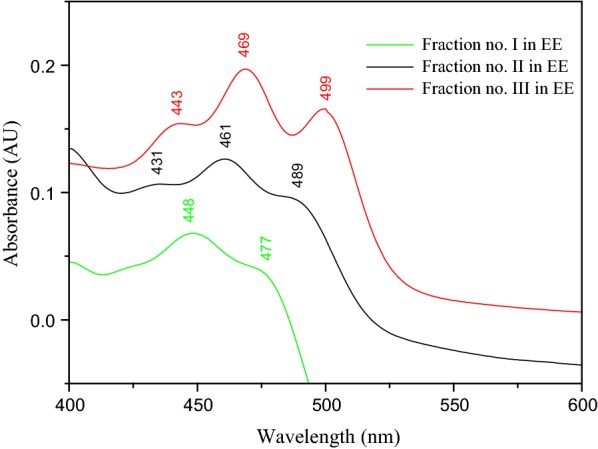
UV–Vis spectra of the TLC separated fractions

As can be seen in Figs. 13 and 14, the maximum absorptions of the β-carotene fractions were obtained at 450 and 448 nm, respectively. These maximum absorptions are very close to those previously reported 448 and 449.5 nm respectively. These maximum absorptions were obtained for β-carotene extracted from the green algae *Chlorococcum humicola* [32].

To determine the structural characteristics of the molecules, absorption spectra have been recorded using the UV/VIS spectrophotometer. The λ_max_ of the absorption spectral bands indicates the length of the p-electron chain and the identity of the chromophore, the carotenoids will absorb at longer wavelengths since have longer systems of linear conjugated double bonds. The absorption spectra for the three major peaks (β-carotene, lutein and lycopene), typical carbonyl-containing carotene and xanthophylls, were broad and devoid of multimodal peaks in the absorption spectrum [32].

## Conclusion

Present results confirmed that *S. limacinum* rich in DHA and *B. trispora* rich in β-carotene, and the variation of their centration value depends on a few factors as temperature, pH and carbon source. The highest concentration of DHA was achieved at 25 °C when the concentration of both carbon sources was 90 g/L. The DHA fraction purity from the total lipid extract after winterization process was 65%. The highest β-carotene production was obtained when the carbon source used was crude glycerol 60 g/L (13.5–14 g/100 wet biomass) at a pH 7. The β-carotene extracted from biomass was purified with a yield higher than 99%. This implies that the glycerol can be utilized as alternative carbon source to glucose in the fermentation medium in order to produce DHA and β-carotene which act as nutraceutical supplements. It appears that glucose was fully utilized in the fermentation medium by the strain, resulting in higher biomass growth but not higher β-carotene or DHA productivity compared with the productivity when using pure or crude glycerol. This could be a potential aspect to offset production costs and enhance commercial viability of the process.

## Abbreviations

*B. trispora*: *Blakeslea trispora*
DHA: docosahexaenoic acid
*S. limacinum*: *Schizochytrium limacinum*
WB: wet biomass DHA
